# Seminal fluid gene expression and reproductive fitness in *Drosophila melanogaster*

**DOI:** 10.1186/s12862-022-01975-1

**Published:** 2022-02-23

**Authors:** Bahar Patlar, Alberto Civetta

**Affiliations:** Department of Biology, University of Winnipeg, Winnipeg, MB R3B 2E9 Canada

**Keywords:** Seminal fluid proteins, Post-mating phenotypes, Sperm competition, Gene expression, RNAi knockdowns

## Abstract

**Background:**

The rapid evolution of seminal fluid proteins (SFPs) has been suggested to be driven by adaptations to postcopulatory sexual selection (e.g. sperm competition). However, we have recently shown that most SFPs evolve rapidly under relaxed selective pressures. Given the role of SFPs in competition for fertilization phenotypes, like the ability to transfer and store sperm and the modulation of female receptivity and ovulation, the prevalence of selectively relaxed SFPs appears as a conundrum. One possible explanation is that selection on SFPs might be relaxed in terms of protein amino acid content, but adjustments of expression are essential for post-mating function. Interestingly, there is a general lack of systematic implementation of gene expression perturbation assays to monitor their effect on phenotypes related to sperm competition.

**Results:**

We successfully manipulated the expression of 16 SFP encoding genes using tissue-specific knockdowns (KDs) and determined the effect of these genes’ perturbation on three important post-mating phenotypes: female refractoriness to remating, defensive (P1), and offensive (P2) sperm competitive abilities in *Drosophila melanogaster*. Our analyses show that KDs of tested SFP genes do not affect female refractoriness to remating and P2, however, most gene KDs significantly decreased P1. Moreover, KDs of SFP genes that are selectively constrained in terms of protein-coding sequence evolution have lower P1 than KDs of genes evolving under relaxed selection.

**Conclusions:**

Our results suggest a more predominant role, than previously acknowledged, of variation in gene expression than coding sequence changes on sperm competitive ability in *D. melanogaster*.

**Supplementary Information:**

The online version contains supplementary material available at 10.1186/s12862-022-01975-1.

## Background

Male biased genes are known to be rapidly evolving, and their rapid evolution has been suggested to be driven by adaptations to reproductive functions [1–3]. Among them, seminal fluid proteins (SFPs) are recognized to be among those evolving the fastest [1, 4–8]. However, both population genetics and molecular evolution studies have found a limited number of SFPs under positive directional selection [4, 7, 9, 10]. A recent population genetics analysis of approximately 300 SFPs using data from a North American and an African population of *Drosophila melanogaster* found, depending on the population assayed, only 7–12% of all known SFPs evolving rapidly under positive selection. Among the remaining SFPs, 35–37% were selectively constrained and 50–57% were selectively relaxed [11].

Functional characterization of SFPs have shown them to be capable to affect reproduction in several ways such as sperm capacity, storage, and release [12, 13], stimulation of female egg production [14, 15], female post-mating physiology and behaviour [16, 17], paternity success and sperm competition [18]. Given the functions of known SFPs in the reproductive success of males by assisting sperm in fertilization, as well as providing defensive and offensive advantages against rivals in sperm competition, the prevalence of relaxed selective pressures shaping the evolution of SFPs is somewhat surprising.

The apparent discrepancy between a large proportion of selectively relaxed SFPs and the known importance of SFPs in reproductive success highlights a long-standing problem of connecting the genotype to the phenotype in an attempt to understand their evolution [19]. This apparent phenotype-genotype disconnection could be attributed to different reasons. One possibility is that a limited number of SFPs under positive or purifying selection are sufficient to drive adaptations at the phenotypic level. This is likely to take place under a genetic architecture in which a handful of major genes exert main effects, with modulation by minor modifiers [20]. For example, in *Drosophila melanogaster*, the nuclear receptor HR39 has been shown as capable of regulating the expression of more than half of putative SFP encoding genes [21]. This genetic architecture predicts that mutations of minor SFP modifiers should have limited effects on fitness. A second possibility is that several SFPs are selectively relaxed because amino acid changes do not cause any modification of protein function (neutral), but changes in expression and the amount of product are essential for gene function and subjected to selective pressures. Indeed, SFPs are highly plastic in gene expression as a response to post-mating selective pressures such as sperm competition [22]. If changes in expression of SFP genes affect male fitness, understanding the effect of gene expression perturbation on fitness shall offer a better opportunity to establish links between the evolution of genotypes and phenotypes.

*Drosophila* females are known to mate multiply with a varying number of males [23, 24]. Thus, while the effect of SFP gene deletions, nulls, and knockdowns in non-competitive mating experiments provide valuable information about gene function, testing the effect of perturbation of gene expression in a design that incorporates competitive mating is critical for an understanding of the role of differential gene expression in sperm competition. Support for a handful of SFPs affecting competitive fitness comes from independent studies that find associations between gene polymorphisms and phenotypes in competitive settings and those that test the effect of the same gene disruption on a phenotype of likely importance in competitiveness (e.g. sperm storage) [18]. There is a lack of systematic implementation of experimental competitive settings and the use of gene perturbation assays to monitor the effect of changes in gene expression on sperm competition phenotypes. A few exceptions are knockdowns of *Acp36DE*, *CG17575*, *CG9997*, and *SP* affecting defensive (P1) and/or offensive (P2) sperm competitive ability [25, 26]. Among these, SP, which is a functionally well-characterized SFP affecting female remating, has been shown as exhibiting high variation in gene expression level that is non-linearly related to female delay to remating [27].

Here we manipulated the expression of 20 SFP encoding genes using tissue-specific knockdowns and determined the effect of successful gene perturbations on important post-mating responses. Specifically, we assayed male fertility in single matings, male paternity success in competition (i.e. defensive and offensive sperm competitiveness; P1 and P2 respectively) [28, 29] and female refractoriness to remate. We selected SFP genes as candidates to test based on (i) evidence from the literature showing associations between SFPs polymorphisms and sperm competitiveness, (ii) SFPs having defined functions in non-competitive sperm physiology or fecundity based on studies that used gene nulls/knockdowns, and (iii) the availability of transgenic *D. melanogaster* strains for gene expression disruption. We also included the SFP gene *Acp29AB* to the list as an internal control, given that it is known to have a significant effect on P1 but not on female refractoriness and P2 [30]. In addition, amongst the genes successfully downregulated, seven genes showed rapid protein coding sequence evolution driven by relaxed selection and five conserved protein coding content due to selective constraints [11]. These genes allowed us to evaluate the effect of gene perturbation of SFPs expression in the context of different evolutionary histories at the protein sequence level.

## Results

### RNAi knockdowns

We tested the effectiveness of knockdowns by using qPCR to measure the expression of target genes (Table 1) in knockdown flies relative to wild-type controls. Out of the 20 SFP genes tested, 13 KDs had significantly lower expression than control males (*P* ≤ 0.1; Additional file 1: Fig. S1). For another 3 genes (*Semp1*, *msopa*, *Qsox4*), expression could not be detected in the KDs (Additional file 1: Fig. S1), but the expression in the control was normal thus indicating nearly complete down-regulation. KD of another four genes (*antr*, *intr*, *CG6168*, *Spn38F*) did not result in a significant down-regulation of gene expression (Additional file 1: Fig. S1).

**Table 1:** SFP genes selected to test their knockdown effect on male fertility, female refractoriness and sperm competitiveness

Gene name	Phenotypes from gene perturbation assays*	Genotype–phenotype associations	Selection regime	References
*Acp29AB*^+^ (CG17797)	P1, sperm storage	P1	Relaxed/Positive	Clark et al. [61], Wong et al. [72]
*Acp33A* (CG6555)		P1, P2	Relaxed	Fiumera et al. [63], Reinhart et al. [78]
*Acp53Ea* (CG8622)		P1	Relaxed	Clark et al. [61]
*Acp76A* (CG3801)		P1, P2	Constrained	Clark et al. [61], Fiumera et al. [60]
*antr* (CG30488)	SP activity, sperm storage, remating, fecundity		Positive	Findlay et al. [64], Singh et al. [65]
*aqrs* (CG14061)	SP activity, sperm storage, remating, fecundity		Constrained	Findlay et al. [64], Singh et al. [65]
*CG11598*	Mediating the activity of AG main cells		Constrained	Hopkins et al. [69]
*CG17242*		Correlated expression with CG9997 +	Relaxed/Positive	Ayroles et al. [70]
*CG34002*		Correlated expression with *CG9997*^+^	Relaxed	Ayroles et al. [70]
*CG9168*		DA in response to density	Constrained	Hopkins et al. [71]
*msopa* (CG14560)		Remating, P1, fecundity	–	Fiumera et al. [60], Zhang et al. [62], Reinhart et al. [78]
*QSox4* (CG31413)		Correlated expression with *CG9997*^+^	Constrained	Ayroles et al. [70]
*Semp1* (CG11864)	Normal processing of genes *Acp26A*^**+**^ and *Acp36DE*^**+**^		Relaxed	Ravi Ram et al. [79], LaFlamme et al. [66]
*Sems* (CG10586)	Sperm storage and release, fecundity		Relaxed	LaFlamme et al. [66]
*Sfp38D* (CG42606)		DA in response to density	Relaxed	Hopkins et al. [71]
*Spn28F* (CG8137)		Remating, P1	Positive	Fiumera et al. [63], Zhang et al. [62]
*Spn77Bc* (CG6289)	Spermatid individualization		Relaxed	Kondo et al. [67]
*Spn38F* (CG9334)		Immune function in females	Relaxed	Mueller et al. [68]
*intr* (CG12558)	SP activity, sperm storage, remating, fecundity		Constrained	Findlay et al. [64], Singh et al. [65]
*CG6168*		Immune function in females, remating, P2	Relaxed/Constrained	Mueller et al. [68], Fiumera et al. [60], Zhan g et al. [62]

*AG* accessory glands, *SP* sex peptide, *DA* differential abundance

^+^Genes known to affect P1 and/or P2 (reviewed in Civetta and Ranz [18])

*All phenotypes, except for Acp29AB, tested in non-competitive assays

### Male fertility in single mating does not decrease in knockdowns

**Fig. 1:**
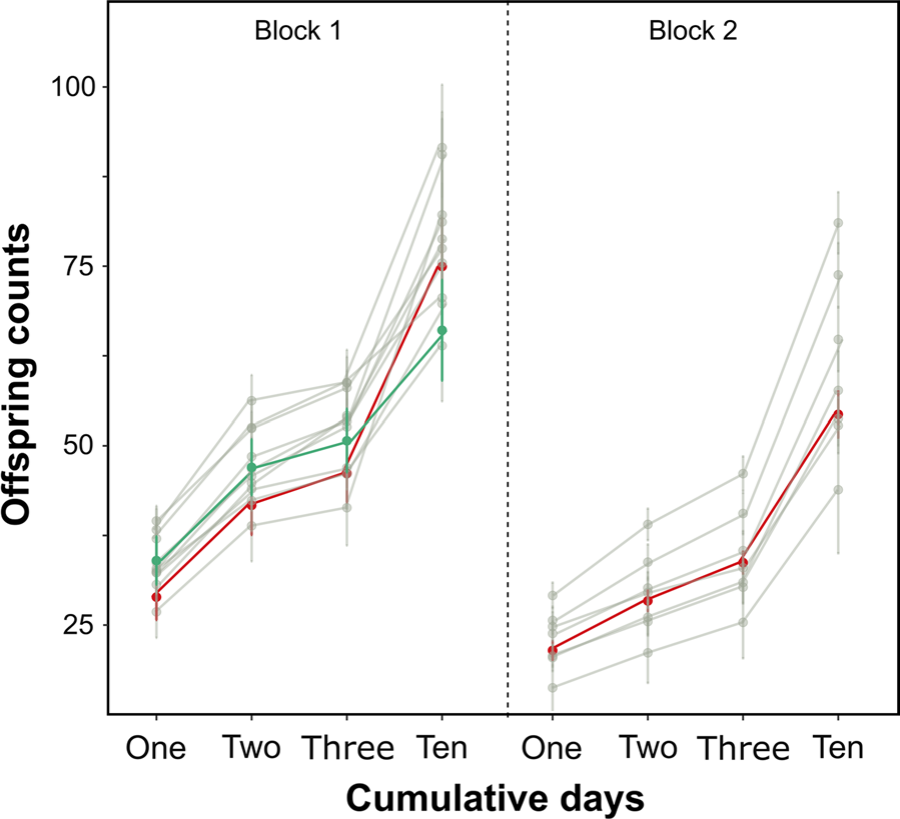
Male fertility for experimental and control males. Mean cumulative number of offspring produced for the first 3 days and at 10 days after mating. Wild-type control (red), GFP reference (green) and SFP knockdowns (grey) are shown. Bars indicate standard errors of the means

Male fertility in single mating assays was scored to test for any significant impairment of the KDs that may affect their competitive paternity in double-mating experiments. Offspring numbers were counted at 1, 2, 3, and 10 days after mating to resemble the intervals used in assays of competitive male paternity. We examined experimental block effects using a two-way ANOVA with days as covariates. There was a significant block effect on offspring counts for the control (F_(1,171)_ = 30.95, *P* < 0.001), therefore we independently tested the fertility differences between KDs and the wild-type control within each experimental replica block. None of the tested males showed a significant decline in average cumulative offspring numbers compared to the control group (One-tailed Welch's *t*-tests; *P* > 0.05) (Additional file 1: Table S2, Fig. 1).

### RNAi knockdown affects sperm competitive ability of first but not second males

**Fig. 2:**
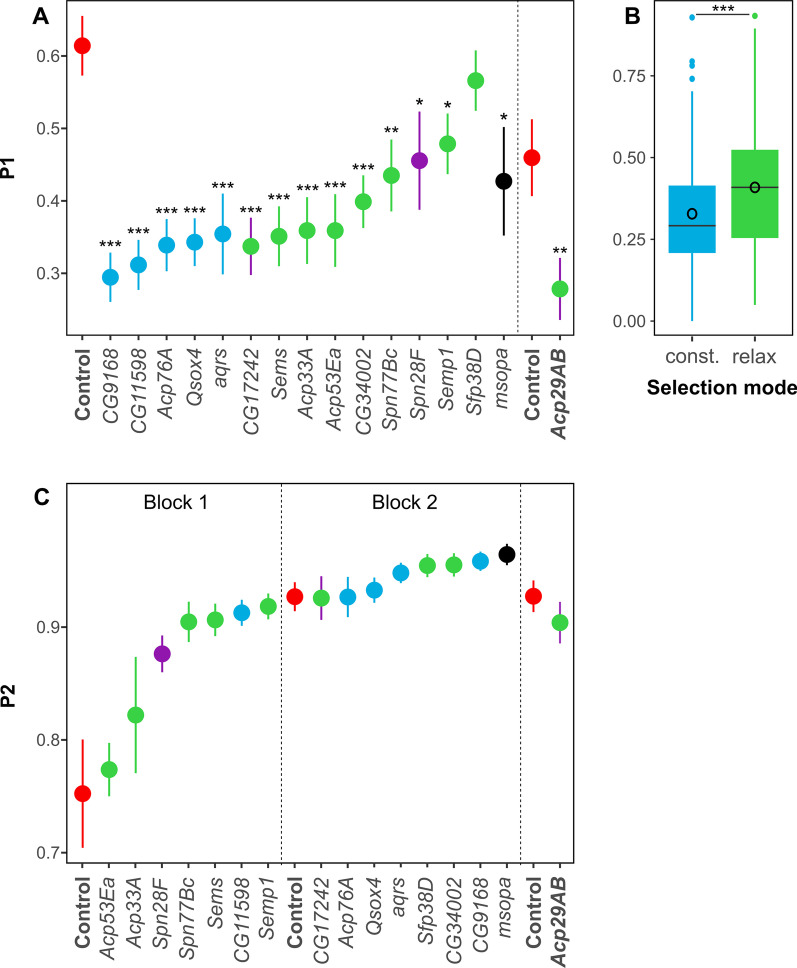
Defensive (P1) and offensive (P2) sperm competitiveness of gene knockdowns and controls. Mean P1 (**A**) and P2 (**C**) for each KD (color coded) and wild-type control (red) are shown with error bars indicating the standard error of the means. Gene KDs are color coded based on their selection regime (Blue: selectively constrained, Green: Relaxed purifying selection, Purple: Positive selection, Black: Unknown). Two genes show both green and purple because they had population specific selection regimes. **B** Boxplot showing differences in overall effect of KDs on P1 for selectively constrained and relaxed genes. The mean and median P1 values are shown as circles and lines within the box, respectively. ***P < .001, **P < .01, *P < .05

We evaluated defensive sperm competitive ability (P1) as short- and long-term relative paternity success (P1 in vial 2 and vial 3, respectively), and overall P1 (relative paternity success over vials 2 and 3). Our comparisons of KDs to the control group for overall P1 showed significant declines in all KDs except for one gene, *Sfp38D* (Fig. 2A, Additional file 1: Table S3). The long-term success of all KDs in P1 (vial 3) was also significantly lower than the control group except for *Sfp38D* (Additional file 1: Table S3). Only eight KDs (*Acp29AB*, *Acp53Ea*, *aqrs*, *CG11598*, *CG34002*, *CG9168*, *Qsox4*, and *Sems*) had significant short-term (vial 2) decline in P1 (Additional file 1: Table S3). Temporal effects of KDs on sperm competitiveness (P1 and P2) have been previously reported [31, 32].

Differences in P1 between males can result from variation in the ability of first to mate males to interfere with the sperm from an incoming second male (e.g. male guardian or copulatory plugs, incapacitation of second male sperm). P1 can also be affected by differences in the properties and functioning of the first male sperm (e.g. differential sperm ejection by females, sperm entry and storage or sperm fertilization ability). Under the first scenario, high P1 values might not associate with the amount of progeny sired by the first male but rather with differences in progeny counts from the second male. We analysed the relationship between the total number of offspring sired by the first male and P1 to evaluate whether the differences in P1 reflect changes in the ability of KD males to assist first male sperm in competition. We found a highly significant regression between offspring number and P1 indicating that variation in P1 can be mostly explained by effects of the SFPs on the properties and functioning of the first male sperm (Adjusted R^2^ = 0.6, *P* < 0.001; Additional file 1: Fig. S2).

Among the 16 genes that we effectively knockdown, the coding sequence evolution of 5 was previously identified as selectively constrained and 7 selectively relaxed [11]. We used these genes to evaluate whether there were differences in the effects of KDs on P1 (phenotype) based on the protein-coding sequences mode of evolution. A one-way ANOVA shows that selectively constrained genes as a group have substantially lower P1 values when their expression is knocked down than group of genes that evolved under relaxed selection (Fig. 2B; F_(1,249)_ = 11, *P* = 0.001).

We found significant differences between the wild-type controls, run in the two separate blocks of P2 experiments, for all sperm competition intervals analysed (F_(1,45)Vial 2_ = 16.7, *P* < 0.001; F_(1,45)Vial 3_ = 6.0, *P* = 0.02; F_(1,45)Vial 2+3_ = 12.8, *P* < 0.001), therefore, results from the two blocks were analyzed separately. We found that all KDs, as well as the internal control gene *Acp29AB*, were non-significantly lower in overall P2 when compared to control wild-type males (Fig. 2C, Additional file 1: Table S4). Short-term (vial 2) and long-term (vial 3) P2 estimates were also non-significant for all gene KDs tested (Additional file 1: Table S4).

### Mates of knockdown males showed no difference in remating compared to controls

**Fig. 3:**
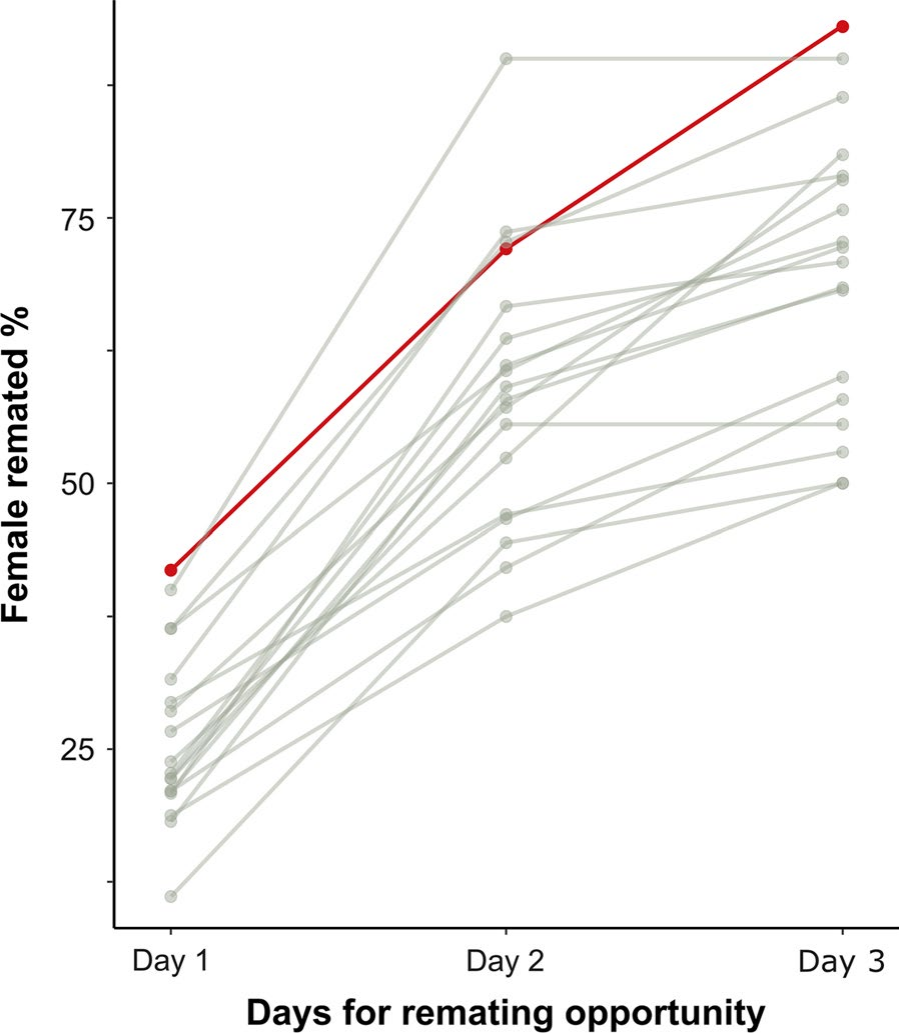
Female refractoriness to remating. The percentage of females that remated with a GFP reference males within 3 days after their first mating with experimental KDs (grey lines) or wild-type control (red line) males

Some SFPs are known to work to make females refractory to remate. Therefore, we expected that at least some of our SFPs KDs would show an increase in remating rates relative to wild-type controls. We found no block effect within any of the days tested (Fisher's exact tests *P* > 0.05), allowing us to pool the blocks for further comparisons. The proportion of females that remated with the reference GFP males after a single mating with a KD was not significantly different than the proportion that remated after mating with a wild-type control (Additional file 1: Table S5, Fig. 3). This result rules out possible effects on sperm competitiveness through manipulation of the females’ mating behaviour.

## Discussion

A handful of functions have been assigned to SFPs through studies that targeted them using gene perturbation and assayed the effect of such manipulation in non-competitive mating trials. The identified functions suggest a role of SFPs in increasing chances for paternity success in defensive situations, such as delay in female remating or enhancing sperm storage and retention [18]. In our study, we showed that down-regulation of 15 tested SFP genes dramatically decreased defensive first male paternity success (P1), while not affecting female refractoriness to remate and second male paternity success (P2). We interpret these results as evidence that SFPs can be more important than previously acknowledged in affecting the ejaculate ability to defend against rival sperm. Moreover, we have recently shown that the protein sequence of the majority of transferred SFPs has evolved under a pattern of relaxed purifying selection [11]. Therefore, our results suggest that gene regulation of SFPs could be an important factor affecting sperm competition phenotypes and that modifications in the amount of SFP produced, rather than changes in the protein itself, might contribute significantly to the evolution of SFP genes.

The last male advantage has been extensively documented in *Drosophila melanogaster* suggesting that defending sperm against upcoming rivals is a critical factor affecting male fitness [24, 33, 34]. We showed that defensive sperm competitive ability (P1) was dramatically decreased in SFPs KDs while male fertility and offensive sperm competitive ability (P2) were unaffected. The block effects we detected in male fertility and P2 tests are not surprising as such effects have been previously found in sperm competition trials and likely result from a myriad of environmental effects [35, 36]. Importantly, the outcome of the gene perturbation effects relative to wild-type controls did not change among blocks.

A male’s sperm defense could be affected by several factors, such as female remating time, sperm storage, ejection, retention, and release. For example, a previous study using null mutant flies characterized the function of the seminal fluid lectin Acp29AB. The absence of Acp29AB strongly decreased P1, but not P2, when females remated 2 days after their first mating. Moreover, females mated with Acp29AB null males were no different in remating rate or fertility. The study showed that Acp29AB is critical in sperm defense because it is needed for sperm retention in storage [30]. Acp36DE nulls affect sperm storage and decrease males’ P1. Interestingly, the effect of Acp36DE nulls on P2 is a consequence of the inability of the sperm of Acp36DE nulls to be retained in storage, and not because Acp36DE is involved in the displacement of resident sperm [25, 37, 38]. There is a limited number of studies that have specifically assayed the effect of gene perturbation of SFPs in competitive settings, but the majority, even when non-competitive settings have been used, identify effects of SFPs on aspects of sperm function such as entry into storage, retention, and release and not in offensive displacement [18]. Our work shows, in a survey of multiple SFPs, that almost all SFPs tested are needed for first male paternity success while not affecting the ability of the males to father progeny when second to mate. We did not test what aspects of sperm competition is disrupted in these knockdowns, but the KDs did not show significant changes in female remating or fertility, suggesting that the decline in P1 is less likely related to changes in female mating behaviour or sperm fertilization capability (i.e. sperm numbers/quality). It could be argued that if roles related to sperm entry, storage and retention are in fact affected in these KDs, P2 should also have been lower for these males. However, the lack of effect in second mating might be explainable if the presence of first wild-type male SFPs in the female is able to help the second KD incoming sperm. A beneficial, rather than detrimental, effect of residual seminal fluids on the sperm of subsequent males has been previously shown in *D. melanogaster* [39, 40].

There are potential limitations to the conclusions we draw about the importance of changes in SFPs expression and P1 that are important to consider. One such limitation is that, for logistic reasons, we compared KDs to a single wild-type control and we used a single reference (GFP) competitor, therefore we lack information on polymorphism and effects on sperm competitiveness. Moreover, we cannot fully rule out the possibility that differences in genetic background of the KD strains might have differentially affected variables like time of maturity or viability that could influence P1 scores. However, we believe such effects are unlikely, as they would have also affected offensive sperm competitiveness (P2). Finally, the use of an AG-specific driver to KD the expression of our target genes might have resulted in an inability to detect effects on reproductive fitness mediated by the genes expression in other tissues. This is highly unlikely for the only gene that did not show any effect on reproductive fitness (Sfp38D) because this gene is AG enriched with almost no expression in other tissues (FlyAtlas2). However, some of the SFPs genes we targeted might exert additional effects on reproductive fitness through their lower but noticeable level of expression in other tissues. For example, some of the genes targeted in our study are reported as having low but detectable expression in the female brain (FlyAtlas2) and it is known that females can influence sperm utilization and both offense and defence sperm competition outcomes [31, 41–43].

The significant effect on phenotype (P1) we observed as driven by the modulation of expression of different SFPs is interesting in the context of the potential role of gene expression in fitness. Previous studies that have not directly manipulated gene expression have also suggested an important role of the amount of SFP gene products in fitness. For example, studies that have modified density to increase the risk and the intensity of sperm competition have found that competitive settings lead to significant adjustment of SFPs expression [44–47]. P1 has been shown to be more sensitive to environmental variation (i.e. normal atmosphere vs. high CO_2_ concentration) than P2 in *Drosophila melanogaster* [48]. Moreover, variation of SFP gene expression generated through genotype-by-environment interactions in competitive and non-competitive settings found a positive relationship between gene expression and P1 but not P2 in flatworms [49]. The use of experimental evolution to enforce monogamy in *Drosophila* has also shown that monogamous males become weaker sperm competitors and have lower expression of SFPs, but do not differ from polygamous males in their ability to diminish female remating [50]. Proteomics has also identified differences in protein expression and abundance of the seminal fluid of primates with different mating systems, with enrichment of proteins with putative roles in the formation of the copulatory plug [51]. Overall, the effect of differential SFPs gene expression in postcopulatory reproductive fitness might be more important than previously acknowledged.

How these changes in expression might shape the long-term evolution of post-mating phenotypes is yet unclear. It is interesting that SFPs we previously identified [11] as evolutionary constrained at the protein sequence level have a more drastic effect on P1, than evolutionary relaxed SFPs, when knockdown. This suggests a positive correlation between protein-coding evolution and gene expression divergence [52–55]. However, studies that have used divergent populations or subspecies have failed to find associations [56, 57]. Moreover, while interesting, we only had a small number of SFPs to compare and to properly establish associations between protein coding mode of evolution and expression of SFPs.

The shifts in expression we induced through gene perturbation are likely detrimental for males because they dramatically lower their fitness (lower P1) relative to wild-type males. Our current result on the effect of changes in expression in fitness, together with our previous identification of a considerable proportion of SFPs with constrained protein sequence evolution [11], raises the question of whether shifts involving relaxation of purifying selection follow by resets of new species-specific patterns of purifying selection might be more relevant than previously considered during the evolution of SFPs. It also remains to be determined whether the genes we have perturbed and assayed in intraspecific settings are also important in the establishment of postcopulatory barriers between species. If so, the finding would add to previous evidence of sperm competition genes contributing to post-mating barriers between species [26, 32, 58] and support the possibility of speciation via sexual selection.

## Materials and methods

### *Drosophila melanogaster* stocks and maintenance

An *Ovulin*-Gal4 driver stock was kindly provided by Dr. M. F. Wolfner (Cornel University, NY, US). Gene-specific UAS-RNAi lines for 20 genes were purchased from the Vienna Drosophila Resource Center (VDRC) or the Bloomington *Drosophila* Stock Center (BDSC). These UAS-RNAi lines are from four different strains (Additional file 1: Table S1) and have different genotypic backgrounds. Wild-type flies were from an isofemale line derived from flies collected in Winnipeg, Manitoba (Canada). A stock that expresses the jellyfish green fluorescent protein (GFP) in the ocelli was purchased from the BDSC (BDSC32170; genotype w[*]; PBac{w[+mW.hs] = GreenEye.UASdsRed}*Dmel*1). Males from this stock were used in competitive paternity assays to identify offspring sired by the common reference GFP male from non-GFP experimental males. Throughout the experiments, flies were kept in either 8 oz. bottles containing 50 ml of cornmeal–yeast–agar–molasses medium (CYAM) or in 27 × 93 mm (diameter × height) vials containing 6–8 ml of medium. Stocks were mantained in a 12:12‐h light–dark cycle at 22 ± 1 °C. Flies were anesthetized using CO_2_ when necessary, but to avoid anaesthetic effects on fitness [59], CO_2_ was never used 24 h prior to experiments. We performed all experiments with three to 5 days old flies.

### Gene selection and RNAi knockdowns

We surveyed the literature to identify candidate SFP genes (Table 1). We first included SFP genes having associations between SNPs and female remating, P1 and/or P2 (*Acp53Ea*, *Acp33A*, *Acp76A*, *CG6168*, *msopa*, *Spn28F*) [60–63]. Second, we included genes having functions in sperm physiology, fecundity or remating based on the effect of knockdowns/nulls or gene ectopic expression (*antr*, *aqrs*, *CG11598*, *intr*, *Semp1*, *Sems*, *Spn77Bc*, *Spn38F*) [64–69]. We also chose three genes (*CG17242*, *CG34002* and *QSox4*) that are genetically correlated in their expression with *CG9997*, a gene known to affect sperm competitiveness [70]. *CG9168* and *Sfp38D* have no known molecular function, but were included because their abundance is dependent on sperm competition intensity [71]. Finally, we also added a control, *Acp29AB*, known to affect P1 [72].

We used an accessory gland tissue specific driver (Ovulin-Gal4) that effectively drives accessory gland-specific expression of target genes in young adult males [73], and all the targeted genes are genes with highly enriched expression in the accessory glands or AG-specific genes (Flyatlas2). To knockdown (KD) expression of these candidate genes, virgin males from UAS-RNAi lines were introduced with virgin females from the *Ovulin*-Gal4 driver strain. Each candidate gene knockdown was replicated in 3–4 vials containing 5 females and 5 males each. To control for larval density, females and males were discarded 5 days after mating. F_1_ virgin males were collected from these replicates, pooled and kept in controlled numbers of 10–15 flies per vial. For verification of the knockdowns, males were chosen randomly from replicates and the male reproductive tract dissected in 1× PBS. For each candidate gene, three RNA biological replicates were prepared from KDs and wild-type control males raised under the same conditions. RNA was isolated by using the Bio-Rad Aurum Total RNA Mini Kit and complementary DNA (cDNA) was synthesized from 1 ml of RNA solution using iScript Select cDNA Synthesis Kit (Bio-Rad, CA, USA) following the manufacturer's instructions. Gene expression was quantified using a BioRad CFX96 Real-Time PCR Detection System. The reactions were performed using the iQ SYBR Green quantitative real-time PCR kit (Bio-Rad, CA, USA). Reaction volumes were set at 10 µl, containing 4 µl iQ SYBR Green Supermix Kit, 150 nM of each primer pair (1.5 µl per pair), 2 µl nuclease‐free water and 1 µl cDNA. Initial thermal cycling conditions were 1 cycle at 95 °C for 5 min, followed by 39 cycles of denaturation at 95 °C for 15 s and annealing at 59 °C for 30 s. Raw Ct values of replicates were obtained from the CFX Software (version 3.0). We designed primers (Additional file 1: Table S1) for each candidate gene using Primer3web version 4.1.0. We have previously tested different reference genes and found that the ribosomal protein *RpS18* had consistently the highest expression and least variability in expression between serial dilution replicas [74]. Thus, we used *RpS18* as a reference gene to normalize gene expression. The expression level of the target gene in each sample was determined by calculating ∆Cq (Cycle quantification) as the Cq of the reference gene (*RpS18*) minus the Cq of the target gene [75].

### Male fertility

Male fertility was measured as the number of offspring fathered by a focal male singly-mated to a virgin wild-type female. The male fertility assays examined whether GFP reference males and knockdowns of SFP genes produced less offspring than wild-type control males. Wild-type females were collected within 3–4 h after emergence and kept in groups of ten for 3–5 days. One day before mating, we housed females singly in freshly prepared vials containing CYAM fly medium and the next morning they were introduced to one same-aged KD, wild-type or GFP male. Pairs were watched until mating occurred for up to three hours. Immediately after mating ended, males were removed, and females were retained for 24 h in the vials to let them lay eggs. We transferred females daily to fresh vials every morning for 3 days (Days 1–3) and then on Day 7, with all females being discarded on Day 10 as female fertility starts to diminish 10 days after mating [76]. We scored offspring and used data to estimate early (Days 1–3) and cumulative (Day 10) male fertility. Male fertility assays were performed in two subsequent blocks, under the exact same conditions, each consisting of the control group (wild-type males) and a subset of KDs.

### Defensive (P1) and offensive (P2) sperm competitive ability

We estimated sperm competitiveness as the proportion of progeny sired by the tested male. A “defense” (P1) and “offense” (P2) score was estimated for each knockdown and control group using GFP males as rivals [18]. In the P1 experiment, we housed virgin females with either KD or control males in bottles containing fresh food (ca. 25–30 individuals from each sex) for about 24 h. After 24 h, we discarded males and transferred females into single vials (Vial 1), where they remain for 2 days to recover mating receptivity. After 2 days, we transferred single females to fresh vials (Vial 2) and introduced one GFP male. GFP males were kept in the Vial 2 for 24 h before being discarded. Females laid eggs in Vial 2 for 2 more successive days and were then transferred to Vial 3 for an additional 4 days. We followed the exact same procedure to measure P2, with tested males (i.e. KDs and wild-type control) being second to mate with females already mated to GFP males. For logistic reasons, we performed P2 experiment in two blocks each containing the control group and a subset of KDs. In addition, both P1 and P2 experiments were conducted separately for the internal control gene, *Acp29AB*.

Females that produced no progeny in Vial 1 were deemed unsuccessful in their first mating and discarded from further analysis. We then counted number of offspring with GFP expression in their ocelli, and those with wild type eyes, using a fluorescent light source under a Nikon SMZ645 dissecting microscope. P1 and P2 were estimated as the proportion of tested males progeny over vials 2 and 3 (overall estimates) as well as in each vial separately (short and long term).

### Female refractoriness to remate

Refractoriness to remate was estimated as the proportion of females that do not remate after a first mating, within a given period of time (24, 48 and 72 h). To estimate refractoriness, virgin wild-type females were first collected and grown under the same conditions as those used in the fertility experiment. Females kept singly housed in vials for 24 h, were presented in the morning with one experimental male (KDs or wild-type control). We observed the pairs until a mating took place or for a maximum of three hrs, at which point males were discarded and females kept in their vials. On the next morning (Day 1), we introduced singly mated females to GFP reference males, and any remating episode was monitored for 7–8 h. Females that did not mate, were given the opportunity to remate with a tester male on subsequent days (Days 2 and 3). The refractoriness experiment was performed in two blocks, under the exact same conditions, each consisting of the control group (wild-type males) and a subset of KDs. For each KD group and control, we counted the number of females remated and not remated to a second GFP male in each day for 3 days in total.

### Statistical analyses

All data was analysed using R-software (v. 4.0.3; [77]). For fertility, P2 and refractoriness, we performed assays in two blocks, with each block consisting of a set of KDs and wild-type control. We first evaluated differences for the estimated phenotypes of controls among the blocks using one-way ANOVA. The data were pooled, if controls did not significantly differ from each other. The knockdown effect on gene expression, fertility and sperm competitiveness (P1 and P2) were assessed using a one-tailed Welch's *t*-test (H_0_: μ_knockdown_ < μ_control_). Refractoriness was tested using a 2 × 2 Fisher Exact tests with KD vs. control as rows and mated or non-mated as columns. *P*-values were adjusted to control for false positives, given multiple tests, using the Benjamini–Hochberg procedure (threshold α = 0.05).

## Supplementary Information

**Additional file 1: Figure S1.** RNAi knockdown efficacy. **Figure S2.** The total number of offspring sired by the first male and its defensive sperm competitiveness, P1. **Table S1.** Gene-specific UAS-RNAi lines for 20 genes and primers used to test the effect of *ovulin* driven (*ovu*-GAL4) downregulation in the accessory glands. **Table S2.** Male fertility tests. **Table S3.** Sperm competitiveness as “defense” ability (P1) of knockdown versus wild-type control males. **Table S4.** Sperm competitiveness as “offense” ability (P2) of knockdown versus wild-type control males. **Table S5.** Female refractoriness tests.

## Data Availability

The datasets generated and/or analyzed during the current study are available in the University of Winnipeg Dataverse repository, at https://doi.org/10.5683/SP3/TBECSY.
