# Supplementary material for: Seminal fluid gene expression and reproductive fitness in Drosophila melanogaster
Source: BMC Ecol Evol. 2022 Feb 23;22:20. doi: 10.1186/s12862-022-01975-1 (PMC8867848; doi:10.1186/s12862-022-01975-1)
Supplement: Supplementary file 1 — Additional file 1: Figure S1. RNAi knockdown efficacy. Figure S2. The total number of offspring sired by the first male and its defensive sperm competitiveness, P1. Table S1. Gene-specific UAS-RNAi lines for 20 genes and primers used to test the effect of ovulin driven (ovu-GAL4) downregulation in the accessory glands. Table S2. Male fertility tests. Table S3. Sperm competitiveness as “defense” ability (P1) of knockdown versus wild-type control males. Table S4. Sperm competitiveness as “offense” ability (P2) of knockdown versus wild-type control males. Table S5. Female refractoriness tests. [file 12862_2022_1975_MOESM1_ESM.docx]

**Additional Tables & Figures**

**Seminal fluid gene expression and reproductive fitness in *Drosophila melanogaster***

Bahar Patlar^1^ & Alberto Civetta^1,2^

^1^Department of Biology, University of Winnipeg, Winnipeg, Manitoba, R3B 2E9

^2^Corresponding author: [a.civetta@uwinnipeg.ca](mailto:a.civetta@uwinnipeg.ca)

**Table S1:** Gene-specific UAS-RNAi lines for 20 genes and primers used to test the effect of *ovulin* driven (*ovu*-GAL4) downregulation in the accessory glands.

|  | **Annotation** | **Gene name** | **Source ID** | **Genotype** | **Primer pairs** | **KD** |
| --- | --- | --- | --- | --- | --- | --- |
| **1** | **CG17797** | *Acp29AB* | BDSC-77372 | y[1] sc[*] v[1] sev[21]; P{y[+t7.7] v[+t1.8]= {TRiP.HMC06058}attP40 | R: TGGAGTTTAAGGCCCAGATG  F: GATGTTGGATGCATGGTGTC | Yes |
| **2** | **CG6555** | *Acp33A* | BDSC-62845 | y[1] sc[*] v[1] sev[21]; P{y[+t7.7] v[+t1.8]= {TRiP.HMC05318}attP40 | R: GCTACCTTCCAAGCGAGTTC  F: CAGTTGTGTGCTGACCCAGT | Yes |
| **3** | **CG8622** | *Acp53Ea* | VDRC-GD52437 | w[1118]; P{GD52437} | R: TGCCAGTCTGCTGAGAGAAA  F: TCCAATATCGGTTGGTGGAT | Yes |
| **4** | **CG3801** | *Acp76A* | BDSC-65182 | y[1] sc[*] v[1] sev[21]; P{y[+t7.7] v[+t1.8]= {TRiP.HMC06056}attP40 | R: AACAAGGTGGCAGTGTCTCA  F: TGGCGAGTACACCATCCAAA | Yes |
| **5** | **CG30488** | *antr* | BDSC-60497 | y[1] v[1]; P{y[+t7.7] v[+t1.8]= {TRiP.HMJ22892}attP40 | R: GCGCCAATACCGACAAGTAT  F: CGCACTTTTAAGGCTCTTGG | Yes |
| **6** | **CG14061** | *aqrs* | BDSC-57197 | y[1] sc[*] v[1] sev[21]; P{y[+t7.7] v[+t1.8]= {TRiP.HMC04579}attP40 | R: GCCTGGTGATCCCTTACTGA  F: GTTGGTGGAGTCGTCATCCT | Yes |
| **7** | **CG14560** | *msopa* | BDSC-64939 | y[1] sc[*] v[1] sev[21]; P{y[+t7.7] v[+t1.8]= {TRiP.HMC05813}attP40 | R: GAAAGGAAGGAGGCCACAATCT  F: CCGCGACTATCAGATACCCTTT | Yes |
| **8** | **CG31413** | *QSox4* | BDSC-60431 | y[1] v[1]; P{y[+t7.7] v[+t1.8]= {TRiP.HMJ22722}attP40 | R: ACCCGCCAACATAAGTGAAG  F: TCCAGATCGGCTCGATAGAT | Yes |
| **9** | **CG10586** | *Sems* | BDSC-52892 | y[1] sc[*] v[1] sev[21]; P{y[+t7.7] v[+t1.8]= {TRiP.HMC03630}attP40 | R: GTTATTGGTGGTCGGGTGAC  F: GATTAAAGTACCGCCGCAGA | Yes |
| **10** | **CG11864** | *Semp1* | VDRC-KK107211 | y,w[1118];P{attP,y[+],w[3`];P{KK107211} | R: GCCAAGTGGGTAAATCTCCA  F: GGCTGCAATACCGTTCACTT | Yes |
| **11** | **CG42606** | *Sfp38D* | BDSC-65974 | y[1] sc[*] v[1] sev[21]; P{y[+t7.7] v[+t1.8]= {TRiP.HMC06254}attP40 | R: CTGGCCCGCTTTTAATGCAA  F: TTTAACCCACCCATGCCACA | Yes |
| **12** | **CG8137** | *Spn28F* | VDRC-KK100958 | y,w[1118];P{attP,y[+],w[3`];P{KK100958} | R: GCCGTCATCTCAAATGGTTT  F: GGCCCTGGAAATAAATGGTT | Yes |
| **13** | **CG6289** | *Spn77Bc* | BDSC-44563 | y[1] sc[*] v[1] sev[21]; P{y[+t7.7] v[+t1.8]= {TRiP.HMS02859}attP40 | R: TTGGCATGGTGCTCAGTTTG  F: AAATTGAAGCCTTGCGTCCG | Yes |
| **14** | **CG11598** |  | BDSC-51000 | y[1] v[1]; P{y[+t7.7] v[+t1.8]= {TRiP.HMJ21113}attP40 | R: CTATTCGTTGCCAGGGTGTT  F: GCGGCAATCAGAGGAATAAC | Yes |
| **15** | **CG17242** |  | BDSC-55198 | y[1] sc[*] v[1] sev[21]; P{y[+t7.7] v[+t1.8]= {TRiP.HMC03896}attP40 | R: ATGCTGCTCAAAGGCATTCT  F: GGAGCTTGTTCAATGCCAAT | Yes |
| **16** | **CG34002** |  | BDSC-61263 | y[1] v[1]; P{y[+t7.7] v[+t1.8]= {TRiP.HMJ23042}attP40 | R: ACAAGTTCAAGGCGAAGGAA  F: TGGATGAAGAAACGTGCTTG | Yes |
| **17** | **CG9168** |  | BDSC-56994 | y[1] sc[*] v[1] sev[21]; P{y[+t7.7] v[+t1.8]= {TRiP.HMC04436}attP40 | R: TGTTCTTCCAACTTCCGGCT  F: TGGCAAAGCGTAGTGAGGTT | Yes |
| **18** | **CG9334** | *Spn38F* | BDSC-41632 | y[1] v[1]; P{y[+t7.7] v[+t1.8]= {TRiP.GL01214}attP40 | R: TACCGGACAAGGTTGATGGT  F: TTGGGAAGCCTTAGATGCAC | No |
| **19** | **CG12558** | *intr* | BDSC-55615 | y[1] sc[*] v[1] sev[21]; P{y[+t7.7] v[+t1.8]= P{TRiP.HMC03754}attP40 | R: CGTCCATCCGATTGATCTCT  F: AACGCAGATGTTGCTGTGAC | No |
| **20** | **CG6168** |  | BDSC-56996 | y[1] sc[*] v[1] sev[21]; P{y[+t7.7] v[+t1.8]= {TRiP.HMC04438}attP40 | R: TGCTGGCAACCGAATCGTAT  F: TGTTCTGTTCTGCTCCGCTT | No |

**Table S2: Male fertility tests.** One-tailed Welch's t-tests were performed to compare the experimental knockdown and reference GFP to the wild-type control males. Fertility was assayed as cumulative average offspring for the first three days and at ten days after mating.

|  |  | **One day** | | | **Two days** | | | **Three days** | | | **Ten days** | | | |
| --- | --- | --- | --- | --- | --- | --- | --- | --- | --- | --- | --- | --- | --- | --- |
| **Block 1** | **N** | **t** | **df** | ***P*** | **t** | **df** | ***P*** | **t** | **df** | ***P*** | **N** | **t** | **df** | ***P*** |
| **GFP** | 17 | -1.10 | 34.35 | 0.86 | -0.93 | 34.97 | 0.82 | -0.77 | 33.92 | 0.77 | 17 | 0.91 | 34.47 | 0.18 |
| ***Acp29AB*** | 18 | -1.90 | 35.91 | 0.97 | -1.94 | 35.97 | 0.97 | -2.06 | 34.67 | 0.98 | 18 | -1.50 | 32.83 | 0.93 |
| ***aqrs*** | 17 | -2.16 | 34.99 | 0.98 | -2.66 | 34.90 | 0.99 | -2.34 | 34.98 | 0.99 | 17 | 0.53 | 33.07 | 0.30 |
| ***CG11598*** | 19 | -0.82 | 36.99 | 0.79 | -0.11 | 36.86 | 0.54 | 0.02 | 35.34 | 0.49 | 19 | 1.07 | 36.04 | 0.14 |
| ***CG17242*** | 19 | 0.43 | 36.14 | 0.34 | 0.44 | 35.44 | 0.33 | 0.72 | 34.05 | 0.24 | 19 | -0.48 | 26.68 | 0.68 |
| ***CG34002*** | 19 | -1.17 | 36.02 | 0.87 | -0.78 | 35.40 | 0.78 | -1.16 | 37.00 | 0.87 | 19 | -0.63 | 36.70 | 0.73 |
| ***CG9168*** | 20 | -2.86 | 31.94 | 1.00 | -2.32 | 28.74 | 0.98 | -2.60 | 29.83 | 0.99 | 19 | -0.29 | 35.36 | 0.61 |
| ***Qsox4*** | 19 | -0.79 | 36.80 | 0.78 | -0.86 | 36.91 | 0.80 | -1.25 | 35.71 | 0.89 | 19 | -0.37 | 35.99 | 0.64 |
| ***Semp1*** | 17 | -0.44 | 33.22 | 0.67 | -0.61 | 33.43 | 0.73 | -1.53 | 34.78 | 0.93 | 17 | -1.73 | 34.95 | 0.95 |
| ***Sems*** | 18 | -0.81 | 35.81 | 0.79 | -0.45 | 34.02 | 0.67 | -0.16 | 34.39 | 0.56 | 18 | 0.56 | 35.98 | 0.29 |
| **Block 2** | **N** | **t** | **df** | ***P*** | **t** | **df** | ***P*** | **t** | **df** | ***P*** | **N** | **t** | **df** | ***P*** |
| ***Acp33A*** | 25 | 0.39 | 46.23 | 0.35 | 1.20 | 43.18 | 0.12 | 1.25 | 41.50 | 0.11 | 25 | 0.12 | 46.18 | 0.45 |
| ***Acp53Ea*** | 24 | 0.44 | 40.75 | 0.33 | 0.83 | 37.50 | 0.21 | 0.86 | 35.20 | 0.20 | 24 | -0.53 | 36.81 | 0.70 |
| ***Acp76A*** | 24 | 1.49 | 30.10 | 0.07 | 1.64 | 28.15 | 0.06 | 1.59 | 27.19 | 0.06 | 24 | 1.11 | 28.81 | 0.14 |
| ***msopa*** | 24 | -3.46 | 41.77 | 1.00 | -4.08 | 39.21 | 1.00 | -4.37 | 38.85 | 1.00 | 24 | -5.01 | 42.40 | 1.00 |
| ***Sfp38D*** | 24 | -1.38 | 38.81 | 0.91 | -0.46 | 40.31 | 0.68 | 0.26 | 40.55 | 0.40 | 24 | 0.30 | 44.30 | 0.38 |
| ***Spn28F*** | 24 | -2.00 | 40.87 | 0.97 | -2.06 | 37.37 | 0.98 | -2.31 | 35.38 | 0.99 | 23 | -3.56 | 40.09 | 1.00 |
| ***Spn77Bc*** | 24 | -1.40 | 41.52 | 0.92 | -1.03 | 37.63 | 0.85 | -0.90 | 34.86 | 0.81 | 23 | -1.91 | 40.12 | 0.97 |

**Table S3:** **Sperm competitiveness as “defense” ability (P1) of knockdown versus wild-type control males.** One-tailed Welch's t-tests were performed to test mean differences for the proportion of offspring sired by knockdown and wild-type control males. *P*-values were adjusted using the Benjamini-Hochberg method. (N: Total number of males tested, M: sample mean, SD: standard deviation of the sample mean). Significant adjusted *P*-values are indicated in bold.

|  |  | Short-term P1 (Vial 2) | | | Long-term P1 (Vial 3) | | | Overall P1 (Vial2+Vial3) | | |
| --- | --- | --- | --- | --- | --- | --- | --- | --- | --- | --- |
|  | **N** | **M±SD** | **t** | ***P*** | **M±SD** | **t** | ***P*** | **M±SD** | **t** | ***P*** |
| Control | 22 | 0.66±0.18 |  |  | 0.56±0.28 |  |  | 0.61±0.19 |  |  |
| *Acp33A* | 20 | 0.58±0.22 | 1.36 | 0.15 | 0.19±0.21 | 5.00 | **<0.001** | 0.36±0.21 | 4.13 | **<0.001** |
| *Acp53Ea* | 19 | 0.44±0.23 | 3.40 | **0.002** | 0.23±0.29 | 3.68 | **<0.001** | 0.36±0.22 | 3.93 | **<0.001** |
| *Acp76A* | 16 | 0.58±0.18 | 1.41 | 0.15 | 0.16±0.16 | 5.57 | **<0.001** | 0.34±0.14 | 5.03 | **<0.001** |
| *aqrs* | 22 | 0.44±0.22 | 3.60 | **0.002** | 0.22±0.31 | 3.76 | **<0.001** | 0.35±0.26 | 3.75 | **<0.001** |
| *CG11598* | 21 | 0.45±0.20 | 3.59 | **0.002** | 0.11±0.15 | 6.56 | **<0.001** | 0.31±0.15 | 5.63 | **<0.001** |
| *CG17242* | 20 | 0.57±0.21 | 1.50 | 0.14 | 0.13±0.17 | 5.82 | **<0.001** | 0.34±0.16 | 4.85 | **<0.001** |
| *CG34002* | 17 | 0.54±0.19 | 2.26 | **0.03** | 0.29±0.22 | 3.56 | **<0.001** | 0.40±0.17 | 3.92 | **<0.001** |
| *CG9168* | 23 | 0.41±0.20 | 4.33 | **<0.001** | 0.21±0.24 | 4.31 | **<0.001** | 0.29±0.15 | 5.98 | **<0.001** |
| *msopa* | 10 | 0.63±0.22 | 0.36 | 0.36 | 0.25±0.27 | 2.92 | **0.006** | 0.43±0.24 | 2.19 | **0.03** |
| *Qsox4* | 21 | 0.55±0.14 | 2.39 | **0.03** | 0.19±0.22 | 4.85 | **<0.001** | 0.34±0.15 | 5.14 | **<0.001** |
| *Semp1* | 21 | 0.61±0.16 | 0.90 | 0.25 | 0.32±0.33 | 2.45 | **0.01** | 0.48±0.18 | 2.31 | **0.02** |
| *Sems* | 19 | 0.44±0.18 | 3.92 | **0.001** | 0.26±0.26 | 3.54 | **0.001** | 0.35±0.18 | 4.51 | **<0.001** |
| *Sfp38D* | 19 | 0.64±0.18 | 0.38 | 0.36 | 0.44±0.29 | 1.31 | 0.10 | 0.57±0.18 | 0.82 | 0.21 |
| *Spn28F* | 15 | 0.62±0.26 | 0.52 | 0.35 | 0.32±0.32 | 2.32 | **0.015** | 0.46±0.26 | 2.00 | **0.03** |
| *Spn77Bc* | 17 | 0.62±0.29 | 0.52 | 0.35 | 0.31±0.23 | 3.01 | **0.003** | 0.44±0.21 | 2.78 | **0.006** |
|  | **N** | **M±SD** | **t** | ***P*** | **M±SD** | **t** | ***P*** | **M±SD** | **t** | ***P*** |
| *Control* | 14 | 0.65±0.15 |  |  | 0.34±0.24 |  |  | 0.46±0.20 |  |  |
| *Acp29AB* | 17 | 0.47±0.23 | 2.61 | **0.007** | 0.15±0.16 | 2.44 | **0.01** | 0.28±0.18 | 2.66 | **0.007** |

**Table S4:** **Sperm competitiveness as “offense” ability (P2) of knockdown versus wild-type control males.** One-tailed Welch's t-tests were performed to test mean differences for the proportion of offspring sired by knockdown and wild-type control males. *P*-values were adjusted using the Benjamini-Hochberg method. (N: Total number of males tested, M: sample mean, SD: standard deviation of the sample mean).

|  |  | Short-term P2 (Vial 2) | | | Long-term P2 (Vial 3) | | | Overall P2 (Vial2+Vial3) | | |
| --- | --- | --- | --- | --- | --- | --- | --- | --- | --- | --- |
| Block 1 | **N** | **M±SD** | **t** | ***P*** | **M±SD** | **t** | ***P*-adj**. | **M±SD** | **t** | ***P*** |
| Control | 23 | 0.64±0.19 |  |  | 0.83±0.34 |  |  | 0.75±0.23 |  |  |
| *Acp33A* | 23 | 0.73±0.22 | -1.56 | 0.94 | 0.89±0.29 | -0.68 | 0.75 | 0.82±0.25 | -0.99 | 0.84 |
| *Acp53Ea* | 22 | 0.61±0.15 | 0.61 | 0.27 | 0.89±0.13 | -0.77 | 0.78 | 0.77±0.11 | -0.40 | 0.65 |
| *CG11598* | 23 | 0.82±0.14 | -3.55 | 1 | 0.98±0.04 | -2.21 | 0.98 | 0.91±0.05 | -3.25 | 1 |
| *Semp1* | 25 | 0.80±0.15 | -3.26 | 1 | 0.99±0.02 | -2.33 | 0.99 | 0.92±0.06 | -3.36 | 1 |
| *Sems* | 25 | 0.81±0.15 | -3.31 | 1 | 0.98±0.03 | -2.23 | 0.98 | 0.91±0.07 | -3.07 | 1 |
| *Spn28F* | 20 | 0.74±0.15 | -1.90 | 0.97 | 0.99±0.02 | -2.35 | 0.99 | 0.88±0.07 | -2.44 | 1 |
| *Spn77Bc* | 24 | 0.80±0.17 | -3.04 | 0.99 | 0.95±0.20 | -1.56 | 0.94 | 0.91±0.09 | -2.97 | 0.99 |
| Block 2 | **N** | **M±SD** | **T** | ***P*** | **M±SD** | **t** | ***P*** | **M±SD** | **t** | ***P*** |
| Control | 23 | 0.84±0.13 |  |  | 0.99±0.01 |  |  | 0.93±0.06 |  |  |
| *Acp76A* | 23 | 0.86±0.13 | -0.52 | 0.70 | 0.97±0.08 | 1.40 | 0.09 | 0.93±0.09 | 0.01 | 0.50 |
| *aqrs* | 23 | 0.88±0.09 | -1.45 | 0.92 | 1.00±0.00 | -1.43 | 0.92 | 0.95±0.04 | -1.34 | 0.91 |
| *CG17242* | 21 | 0.85±0.16 | -0.44 | 0.67 | 0.97±0.07 | 1.52 | 0.07 | 0.93±0.09 | 0.05 | 0.48 |
| *CG34002* | 20 | 0.90±0.10 | -1.90 | 0.97 | 0.99±0.01 | 0.09 | 0.46 | 0.96±0.05 | -1.71 | 0.95 |
| *CG9168* | 23 | 0.91±0.10 | -2.04 | 0.98 | 0.99±0.02 | 0.66 | 0.26 | 0.96±0.04 | -2.04 | 0.98 |
| *msopa* | 23 | 0.91±0.11 | -2.26 | 0.99 | 1.00±0.00 | -1.43 | 0.92 | 0.96±0.05 | -2.34 | 0.99 |
| *Qsox4* | 26 | 0.84±0.14 | -0.21 | 0.58 | 0.99±0.01 | 0.36 | 0.36 | 0.93±0.06 | -0.34 | 0.63 |
| *Sfp38D* | 25 | 0.91±0.10 | -2.13 | 0.98 | 0.99±0.01 | 0.47 | 0.32 | 0.96±0.05 | -1.68 | 0.95 |
|  | **N** | **M±SD** | **t** | ***P*** | **M±SD** | **t** | ***P*** | **M±SD** | **t** | ***P*** |
| Control | 19 | 0.80±0.15 |  |  | 0.99±0.01 |  |  | 0.93±0.06 |  |  |
| *Acp29AB* | 19 | 0.77±0.17 | 0.59 | 0.28 | 0.97±0.08 | 1.39 | 0.09 | 0.90±0.08 | 1.01 | 0.16 |

**Table S5: Female refractoriness tests.** Fisher Exact tests were performed to compare knockdown and control groups for the proportion of females remated or not remated within 24, 48, and 72 hrs. after a single mating. (N: Number of females remated / Number of females that did not remate).

|  | 24 hrs. | | | 48 hrs. | | | 72 hrs. | | |
| --- | --- | --- | --- | --- | --- | --- | --- | --- | --- |
|  | **N** | **Odds** | ***P*** | **N** | **Odds** | ***P*** | **N** | **Odds** | ***P*** |
| Control | **18/25** |  |  | **31/12** |  |  | **40/3** |  |  |
| *Acp29AB* | **3/13** | 3.06 | 0.98 | **6/10** | 4.19 | 1 | **8/8** | 12.52 | 1 |
| *Acp33A* | **5/19** | 2.70 | 0.98 | **16/8** | 1.29 | 0.77 | **17/7** | 5.34 | 1 |
| *Acp53Ea* | **5/16** | 2.27 | 0.96 | **11/10** | 2.31 | 0.97 | **17/4** | 3.07 | 0.97 |
| *Acp76A* | **4/10** | 1.78 | 0.89 | **8/6** | 1.91 | 0.92 | **11/3** | 3.54 | 0.97 |
| *aqrs* | **8/12** | 1.08 | 0.66 | **18/2** | 0.29 | 0.10 | **18/2** | 1.47 | 0.82 |
| *CG11598* | **6/13** | 1.55 | 0.85 | **14/5** | 0.92 | 0.58 | **15/4** | 3.47 | 0.98 |
| *CG17242* | **5/12** | 1.71 | 0.88 | **8/9** | 2.85 | 0.98 | **9/8** | 11.19 | 1 |
| *CG34002* | **4/14** | 2.48 | 0.96 | **10/8** | 2.04 | 0.94 | **10/8** | 10.13 | 1 |
| *CG9168* | **4/15** | 2.66 | 0.97 | **8/11** | 3.47 | 1 | **11/8** | 9.23 | 1 |
| *msopa* | **4/18** | 3.18 | 0.99 | **13/9** | 1.77 | 0.91 | **15/7** | 6.02 | 1 |
| *Qsox4* | **2/16** | 5.61 | 1 | **8/10** | 3.16 | 0.99 | **9/9** | 12.57 | 1 |
| *Semp1* | **4/15** | 2.66 | 0.97 | **11/8** | 1.86 | 0.92 | **13/6** | 5.94 | 1 |
| *Sems* | **4/14** | 2.48 | 0.96 | **11/7** | 1.63 | 0.87 | **13/5** | 4.96 | 0.99 |
| *Sfp38D* | **12/21** | 1.26 | 0.76 | **20/13** | 1.67 | 0.90 | **25/8** | 4.18 | 0.99 |
| *Spn28F* | **8/14** | 1.26 | 0.75 | **16/6** | 0.97 | 0.60 | **19/3** | 2.08 | 0.90 |
| *Spn77Bc* | **5/17** | 2.42 | 0.97 | **14/8** | 1.47 | 0.84 | **16/6** | 4.86 | 0.99 |

**Figure S1 – RNAi knockdown efficacy.** One-tailed two samples Welch's t-tests were performed comparing the average relative expression level of target genes in wild-type control and RNAi knockdown. Significant *P*-values are in bold (P≤ 0.1).


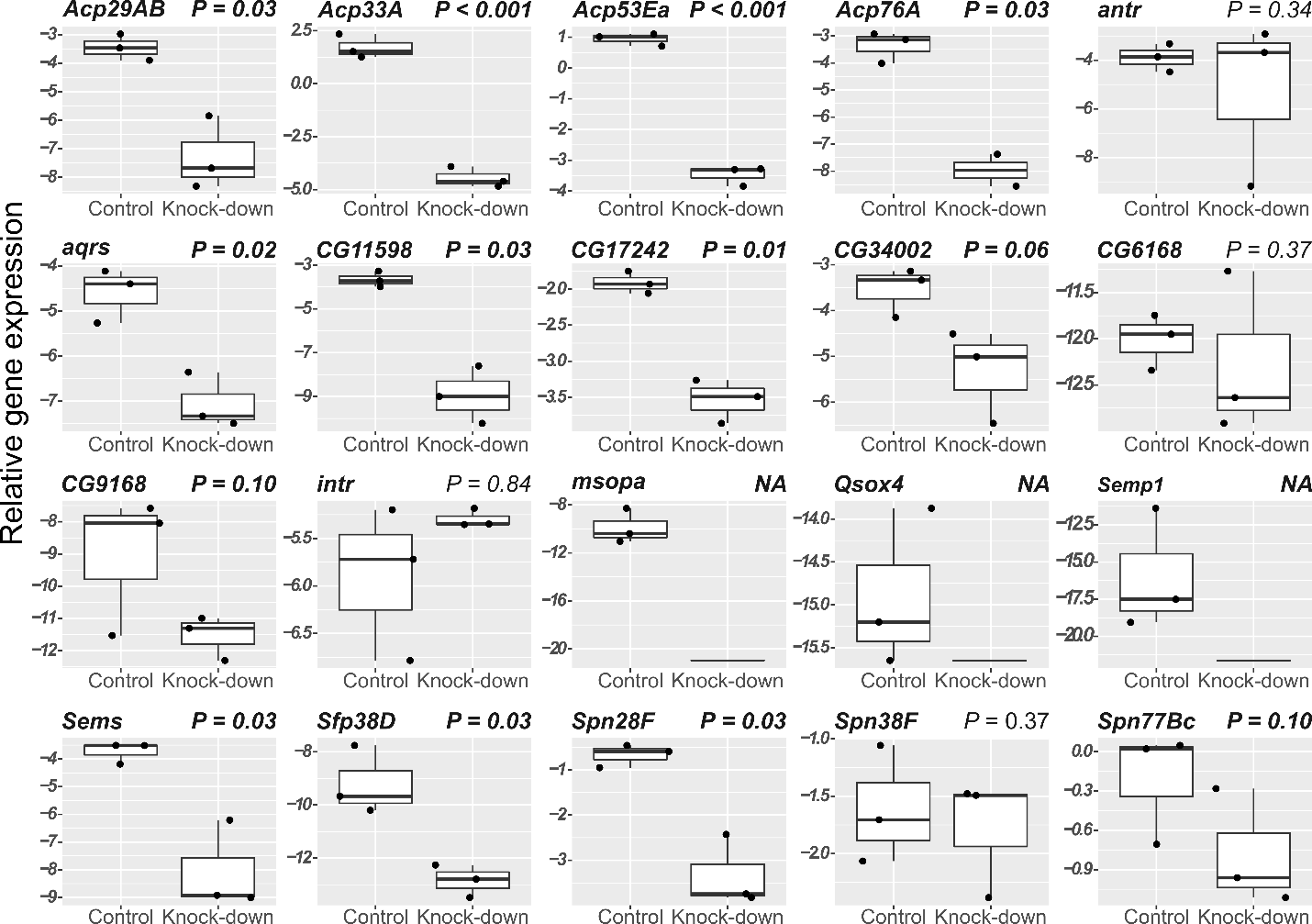


**Figure S2: The total number of offspring sired by the first male and its defensive sperm competitiveness, P1.** Scatters show each individual sample from gene KDs and controls. The red line is the linear regression model fit with the grey shaded areas indicating the 95% confidence interval.

**
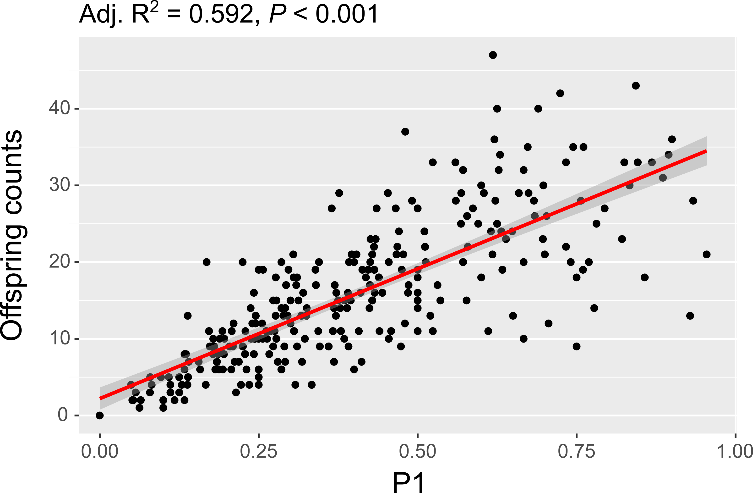
**
